# The small GTPase RAB10 regulates endosomal recycling of the LDL receptor and transferrin receptor in hepatocytes

**DOI:** 10.1016/j.jlr.2022.100248

**Published:** 2022-06-24

**Authors:** Taslima Gani Khan, David Ginsburg, Brian T. Emmer

**Affiliations:** 1 Program in Chemical Biology, University of Michigan, Ann Arbor, Michigan, USA; 2 Life Sciences Institute, University of Michigan, Ann Arbor, Michigan, USA; 3 Department of Internal Medicine, University of Michigan, Ann Arbor, Michigan, USA; 4 Howard Hughes Medical Institute, University of Michigan, Ann Arbor, Michigan, USA; 5 Departments of Human Genetics and Pediatrics, University of Michigan, Ann Arbor, Michigan, USA

**Keywords:** low density lipoprotein receptor, receptors, protein trafficking, cholesterol, lipoproteins, CRISPR screen, HuH7 cells, endocytosis, RAB10, RAB11, ERC, endocytic recycling compartment, LDLR, LDL receptor, TFR, transferrin receptor

## Abstract

The low-density lipoprotein receptor (LDLR) mediates the hepatic uptake of circulating low-density lipoproteins (LDLs), a process that modulates the development of atherosclerotic cardiovascular disease. We recently identified *RAB10*, encoding a small GTPase, as a positive regulator of LDL uptake in hepatocellular carcinoma cells (HuH7) in a genome-wide CRISPR screen, though the underlying molecular mechanism for this effect was unknown. We now report that RAB10 regulates hepatocyte LDL uptake by promoting the recycling of endocytosed LDLR from RAB11-positive endosomes to the plasma membrane. We also show that RAB10 similarly promotes the recycling of the transferrin receptor, which binds the transferrin protein that mediates the transport of iron in the blood, albeit from a distinct RAB4-positive compartment. Taken together, our findings suggest a model in which RAB10 regulates LDL and transferrin uptake by promoting both slow and rapid recycling routes for their respective receptor proteins.

An elevated level of circulating low-density lipoprotein (LDL) cholesterol is a major risk factor for atherosclerotic cardiovascular diseases, including myocardial infarction and stroke (1, 2, 3, 4, 5, 6, 7). Regulation of plasma cholesterol is governed by a complex interplay between dietary absorption, de novo biosynthesis, and clearance from the bloodstream. Therapeutic targeting of LDL clearance has been a highly successful strategy for the prevention and treatment of atherosclerosis. LDL clearance is mediated by the LDL receptor (LDLR), a cell-surface glycoprotein that directly binds to the apolipoprotein B component of LDL particles and triggers clathrin-mediated endocytosis. The acidic environment of the endosomal lumen induces complex dissociation, with LDL subsequently transported to the lysosome for hydrolysis, and free LDLR recycled back to the plasma membrane (8, 9). Many regulatory proteins affecting the endocytic pathway and cell-surface expression of LDLR have been identified, including PCSK9, a negative regulator that redirects LDLR to the lysosome for degradation (10), and IDOL, a ubiquitin ligase that induces proteasomal degradation of LDLR (11, 12). Although much is known about the regulation of LDLR expression and endocytosis, questions remain concerning the molecular determinants of LDLR recycling.

We recently reported a genome-wide CRISPR screen for modifiers of LDL uptake in HuH7 cells (13). This screen identified RAB10, a small GTPase known to mediate trafficking of vesicles between intracellular compartments, as a key regulator of LDL uptake. Deletion of *RAB10* decreased cellular endocytosis of LDL but increased accumulation of another endocytic cargo, transferrin. The receptors for LDL (LDLR) and transferrin receptor (TFR) are both endocytosed from the cell surface via clathrin-coated vesicles and transported through intracellular recycling pathways (14, 15, 16, 17, 18, 19, 20). In this study, we investigated the role of RAB10 in LDL and transferrin endocytosis. Our results demonstrate that GTP-bound RAB10 positively regulates the activity of LDLR and TFR by accelerating the recycling of both proteins to the plasma membrane.

## Materials and methods

### Antibodies

**Table:** 

Antibody	Supplier /ID	Experiment	Dilution
Anti LDLR	Proteintech; 10785-1-AP	Western blot	1:2000
Anti LDLR	Santa Cruz biotechnology; sc-18823(clone C7)	Immunofluorescence Endocytosis assay	1:400 1.25ul(0.25ug)/100ul
Anti LDLR	R & D system; FAB2148G Alexa488	Protein quantification by FACS	5ul/100ul(10^6^ cells)
Anti TFR	Santa Cruz biotechnology; sc-65882/H68.4	Western blot Immunofluorescence	1:1000 1:400
Anti TFR-FITC	Fisher/11-0719-42 (clone OKT9)	Protein quantification by FACS	5ul(0.125ug)/100ul(10^5^–10^8^ cells/100ul)
Anti Rab10	Abcam/ab237703	Western blot Immunofluorescence	1:1000 1:400
Rabbit anti EEA1	Cell Signaling Technology, Inc/3288S(clone C45B10)	Immunofluorescence	1:100
Mouse anti EEA1	BD BIOSCIENCE /610,456	Immunofluorescence	1:400
Rabbit Anti Rab11	Cell Signaling Technology /(clone D4F5) 5589S	Immunofluorescence	1:100
Mouse anti Rab11	Santa Cruz biotechnology/(clone D-3) sc-166523	Immunofluorescence	1:50
Rabbit anti LAMP1	Santa Cruz biotechnology /9091S	Immunofluorescence	1:100
Rabbit PDI	Cell Signaling Technology /3501S	Immunofluorescence	1:100
Mouse anti PDI	Thermo Scientific/MA3-019	Immunofluorescence	1:50
Rabbit TGN46	Abcam/ab50595	Immunofluorescence	1:100
Sheep anti Human TGN46	Bio-Rad/AHP500GT	Immunofluorescence	1:200
Rabbit anti GM130	Abcam/ab52649	Immunofluorescence	1:100
Rabbit anti Rab7(D95F2)	Cell Signaling Technology, Inc/ 9367T	Immunofluorescence	1:100
Donkey anti-Sheep IgG-Alexa Fluor 488	Thermo Scientific / A-11015	Immunofluorescence	1:500
Donkey anti Mouse IgG-Alexa Fluor 488	Thermo Scientific / A-21202	Immunofluorescence FACS	1:1000
Donkey anti Rabbit IgG-Alexa Fluor Plus 594	Fisher/ A32754	Immunofluorescence	1:500
β-Actin Antibody (C4)	Santa Cruz biotechnology /sc-47778	Western blot	1:1000
Anti mouse		Western blot	1:5000
Anti rabbit		Western blot	1:5000

### Oligonucleotide sequences

**Table:** 

gRNA	Sequence
*RAB10* gRNA	TGATGGTGTGAAATCGCTCC
*LDLR* gRNA	AACAAGTTCAAGTGTCACAG
*TFRC* gRNA	CGGTAGACTTGTTTACCTGG
Non targeting gRNA	CGTGTGTGGGTAAACGGAAA

### Plasmid, virus, and cell culture

For CRISPR-mediated gene knockouts, the sgRNA sequences were cloned into the CRISPR plasmid pLentiCRISPRv2(Addgene, MA #52961) as previously described (13). Virus particles were then prepared by cotransfection of cloned sgRNA together with pCMV-VSV-G (Addgene #8454) and (Addgene #12260) into HEK293T cells with Lipofectamine LTX (ThermoFisher). Media were replaced at 12 h post transfection. Conditioned media containing virus were harvested at 48 h posttransfection, centrifuged at 1000g for 10 min, and the resulting supernatant stored at −80 C for future use. To generate knockout cells, HuH7 cells were transduced with lentivirus carrying the corresponding sgRNA, selected for transduced cells with puromycin, and passaged for two weeks to allow time for target site mutagenesis and turnover of wild-type protein. RAB10 knockout clonal cell lines were derived by diluting cell suspensions into 96-well plates. Wells containing a single colony of growth were then expanded. Selected clonal cell lines were analyzed by immunofluorescence and immunoblotting. RAB10 expression constructs were generated by cloning CRISPR-resistant cDNA sequences and a blasticidin-resistance cassette into the lentiviral expression vector LeGO-iC2(Addgene, 27,345) using GIBSON assembly mix purchased from NEB (NEBuilder HiFi DNA Assembly). HEK293T and HuH7 cells were cultured in Dulbecco’s modified Eagle’s medium (DMEM) supplemented with 10% fetal bovine serum, 100 U/ml penicillin, and 100 mg/ml streptomycin (Thermo Scientific) at 37°C in a 5% CO2-conditioned, humidified incubator.

### LDL and transferrin uptake assay

Cells were seeded in 6-well plates to achieve ∼70%–80% confluence on the day of analysis. For uptake assays, cells were washed with serum-free DMEM and then incubated in DMEM containing either 4 μg/ml DyLight550-conjugated LDL (Cayman Chemical) or 5 μg/ml Alexa Fluor 555-conjugated transferrin (ThermoFisher Scientific) at 37°C for 1 h or 30 min, respectively. Cells were harvested with TrypLE express (ThermoFisher Scientific), washed with ice cold PBS, resuspended in 150ul of ice-cold PBS, and analyzed with a Bio-Rad Ze5 flow cytometer. Data analysis was performed with FlowJo (FlowJo).

### Western blot

Cells were cultured at 37°C in 10 cm dish until 70%–80% confluent. Cells collected with trypLE express were washed in PBS and then lysed in RIPA lysis and extraction buffer (Thermo Scientific) containing complete protease inhibitor cocktail (Roche). After brief sonication, lysed cell suspensions were rotated at 4°C for 1 h for protein extraction followed by centrifugation at 15000g. Protein concentration was determined with the Bio-Rad DC assay kit (Bio-Rad, # 500-0111), and SDS-PAGE was performed using NuPAGE™ 4%–12%, Bis-Tris, mini protein gels (ThermoFisher Scientific # NP0321BOX) according to manufacturer’s instruction. Western blot transfer was done into nitrocellulose membrane (Thermo Scientific #IB23002) using the iBlot 2 Dry Blotting System (Thermo Scientific).

### Flow cytometry

HuH7 cells cultured in 6-well plates were prepared for analysis at 70%–80% confluence. For surface staining, collected cells were washed three times with ice-cold blocking buffer (PBS, 2% FBS), resuspended at approximately 10^6^ cells in 1 ml blocking buffer and incubated for 30 min with end-over-end rotation at 4°C. After centrifugation at 400g for 5 min, cells were resuspended in fluorescently labeled LDLR antibody or TFR antibody diluted in 100 μl blocking buffer and incubated for 1 h in the dark at 4°C. Cells were then washed three times with ice-cold PBS, resuspended in 150 μl cold PBS for final analysis by flow cytometry (Bio-Rad ZE5). For quantification of total cellular LDLR or TFR, harvested cells were fixed with 2% PFA for 10 min followed by PBS wash and permeabilization with 500 μl of 0.5% saponin in PBS before proceeding with staining for LDLR and TFR.

### Immunofluorescence and confocal microscopy

Cells cultured on poly-D-lysine–coated glass coverslips (Electron Microscopy Sciences, #72294-11) were fixed in 2% paraformaldehyde for 15 min in the dark at room temperature. After washing three times with PBS, cells were then permeabilized with 0.1% saponin in PBS for 5 min, incubated for 1 h in blocking buffer (PBS with 4% FBS and 40 mM glycine), stained with primary antibody at indicated dilutions in PBS with 4% FBS for 1 h, washed with PBS three times, stained with secondary antibody at indicated dilutions in PBS with 4% FBS for 1 h, and washed with PBS three times. Coverslips were mounted on glass slides with ProLong Diamond antifade mounting reagent (Invitrogen). Images were acquired with a NIKON A1 standard sensitivity confocal microscope with 60X (NA51.4) oil objective. Colocalization quantification was done using the open-source Fiji (Image J) software. Mander's coefficient and Pearson's coefficient were calculated using JACop in Image J. A total of 10–30 cells from 2 to 3 biological replicates were analyzed. For all quantitative analysis, the observer was blinded to cell genotype.

### Endocytosis assay

An assay for transferrin receptor endocytosis was adapted from previous reports (21). Briefly, cells grown in 10 cm dishes were serum starved in DMEM for 30 min, harvested in trypLE Express (ThermoFisher Scientific), washed in ice cold DMEM, incubated with Alexa Fluor 555-conjugated transferrin in DMEM at 4°C, and rotated for 1 h. Unbound excess transferrin was removed by washing cells with PBS, and surface-bound transferrin internalization was induced by incubating cells in prewarmed complete culture medium at 37°C for various time points. At each time point, an excess of ice-cold PBS was added to a sample to stop internalization, cells were collected by centrifugation, and surface-bound transferrin was removed with ice-cold acid wash buffer (0.1 M glycine and 150 mM NaCl, pH 3) followed by three PBS washes. Cells were resuspended in ice-cold PBS and analyzed by flow cytometry on a Bio-Rad ZE5. 10,000–15,000 cells were analyzed for each time point.

An assay for LDLR endocytosis was adapted from previous reports (22, 23). Briefly, after PBS wash, cells were incubated in blocking buffer (2% FBS in PBS) for 30 min at 4°C. Surface LDLR was then stained with LDLR antibody for 1 h at 4°C, and cells were washed with PBS to remove excess antibody. Cells were then incubated with prewarmed media at 37°C for the indicated duration of time. At each time point, ice-cold blocking buffer was added to the sample, cells were collected by centrifugation, and the remaining surface-exposed LDLR antibody was labeled by incubation with fluorescent secondary antibody for 1 h at 4°C followed by three PBS washes. Cells were resuspended in ice-cold PBS. Analysis was performed by flow cytometry on a Bio-Rad ZE5, with 10,000–15,000 cells analyzed for each time point.

### Recycling assay

An assay for transferrin recycling was adapted from a previous report (21). Briefly, cells were serum starved for 30 min in DMEM, incubated with Alexa Fluor 555 transferrin for 30 min at 37°C, and washed with ice-cold PBS. Surface-bound transferrin was then removed by cold acid wash (0.1 M glycine and 150 mM NaCl, pH 3) followed by a PBS wash. Cell samples were resuspended in prewarmed media at 37°C for the indicated times. A second acid wash followed by PBS wash was done after which samples were analyzed by flow cytometry.

## Results

### RAB10 regulates the cellular uptake of LDL and transferrin

**Fig. 1:**
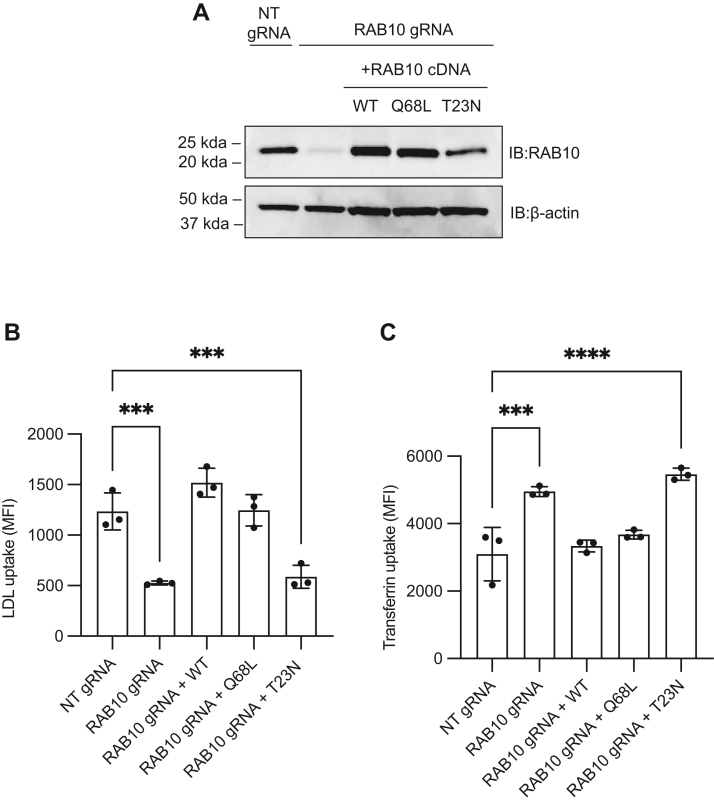
RAB10 exhibits opposite effects on cellular accumulation of LDL and transferrin. A: Immunoblotting of lysates prepared from HuH7 cells treated with a control nontargeting (NT) gRNA or a gRNA targeting *RAB10*, with or without heterologous expression of a CRISPR-resistant wild-type (WT), GTP-locked (Q68L), or GDP-locked (T23N) *RAB10* cDNA. B–C: Fold change of internalized fluorescent LDL (B) or transferrin (C) relative to NT control for cells indicated in (A). Individual data points represent independent biologic replicates, error bars indicate standard deviation. ^∗∗∗^*P* value < 0.0005; ∗∗∗∗ *P* value < 0.0001 (one-way ANOVA test).

To test the influence of RAB10 on LDL uptake, we generated RAB10-deficient HuH7 cells by CRISPR-mediated disruption of the *RAB10* gene and confirmed efficient depletion of RAB10 protein by immunoblotting (Fig. 1A). Consistent with the findings of our previous CRISPR screen (13), *RAB10*-targeted cells exhibited decreased uptake of fluorescently labeled LDL and increased accumulation of fluorescently labeled transferrin relative to control cells treated with a nontargeting gRNA (Fig. 1B, C). To rule out a CRISPR off-target effect, we transduced *RAB10*-targeted cells with a lentivirus directing expression of *RAB10* cDNA with a synonymous mutation conferring resistance to CRISPR disruption; this heterologous expression of wild-type *RAB10* cDNA rescued LDL and transferrin uptake, confirming that the observed effects of *RAB10*-targeting were mediated by on-target activity (Fig 1B, C). We also tested the requirement of GTPase cycling for RAB10 function in LDL and transferrin uptake by expressing RAB10 point mutants locked in the GTP-bound or GDP-bound states (Fig. 1A) (24, 25, 26, 27). Expression of both wild-type and GTP-locked RAB10 (Q68L) rescued the LDL (Fig. 1B) and transferrin (Fig. 1C) uptake phenotype of *RAB10*-targeted cells, whereas expression of GDP-locked RAB10 (T23N) had no effect.

### RAB10 regulates the cellular distribution of LDLR and TFR

**Fig. 2:**
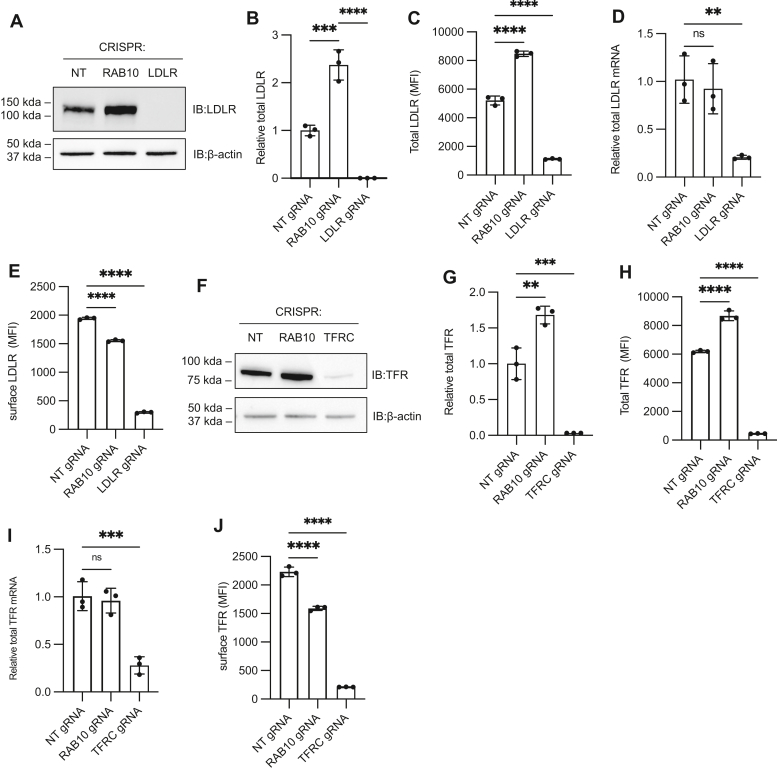
RAB10 regulates cell-surface expression of LDLR and TFR. HuH7 cells were treated with a gRNA targeting *RAB10*, *LDLR*, *TFRC*, or a nontargeting (NT) control. Changes in LDLR (A–E) and TFR (F–J) were evaluated by Western blotting (A-B, F-G) or flow cytometry of permeabilized cells (C, H) for total cellular protein, flow cytometry of intact cells for surface-displayed protein (E, J), or qRT-PCR for mRNA abundance (D, I). Individual data points represent independent biologic replicates, error bars indicate standard deviation. ∗∗*P* value < 0.005; ∗∗∗*P* value < 0.0005; ∗∗∗∗*P* value < 0.0001 (one-way ANOVA test). LDLR, LDL receptor; TFR, transferrin receptor.

To clarify the molecular basis for altered LDL and transferrin uptake in RAB10-deficient cells, we analyzed the total protein abundance and surface expression for the corresponding cellular receptors, LDLR (28) and TFR (19). Despite the decreased LDL uptake observed in *RAB10*-targeted cells (Fig. 1B), these same cells exhibited increased levels of total cellular LDLR protein, as measured by both immunoblotting (Fig. 2A, B) and by flow cytometry of permeabilized cells (Fig. 2C), with no corresponding change in *LDLR* transcript levels by qRT-PCR (Fig. 2D). This increase in total cellular LDLR protein but not mRNA in *RAB10*-targeted cells suggested that the observed decrease in LDL uptake by these cells was due to a defect in either LDLR trafficking or activity rather than an effect on *LDLR* gene expression. Indeed, flow cytometry of nonpermeabilized *RAB10*-targeted cells demonstrated a redistribution of cellular LDLR protein from the cell surface to intracellular compartments (Figs. 2E and S1A). In contrast, overexpression of either wild-type or GTP-locked RAB10 had the opposite effect, increasing the proportion of LDLR at the cell surface (supplemental Fig S1B).

To examine the effects of RAB10 on TFR, we similarly analyzed the distribution of TFR in *RAB10*-targeted cells. Despite the divergent effects of *RAB10*-targeting on LDL and transferrin uptake (Fig. 1B, C), the effect on TFR abundance mirrored its effect on LDLR, with total cellular TFR protein levels increased (Fig. 2F–H), while mRNA levels were unchanged (Fig. 2I), and surface-displayed protein levels were reduced (Fig. 2J). These similarities suggest that the discordant effects of *RAB10* targeting on LDL and transferrin cellular accumulation are a result of different fates of the labeled ligand rather than their corresponding receptors. Consistent with this interpretation, internalized LDL is released from LDLR in acidic compartments (29, 30) whereas transferrin remains in complex with TFR until it is released to the extracellular environment after TFR is recycled back to the plasma membrane (18, 20).

### Heterogeneous distribution of RAB10 in subcellular compartments

**Fig. 3:**
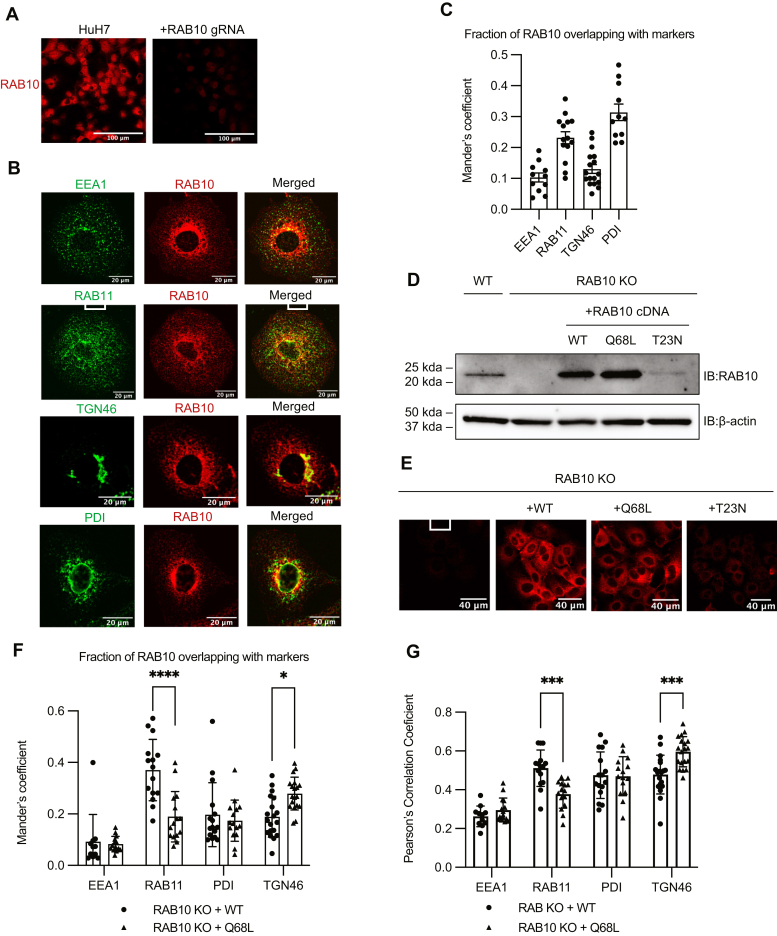
Characterization of subcellular localization of RAB10. A: RAB10 immunofluorescence of wild-type and *RAB10*-targeted HuH7 cells. B: Costaining of wild-type HuH7 cells for RAB10 and the indicated markers for different subcellular compartments. C: Mander’s coefficient showing overlap of endogenous RAB10 with the indicated marker in HuH7 cells. D: Western blot of lysates prepared from wild-type and a *RAB10*-deleted HuH7 clonal cell line with or without heterologous expression of wild-type, Q68L, or T23N CRISPR-resistant *RAB10* cDNA. E: RAB10 immunofluorescence of the cells indicated in (D). F–G: Mander’s coefficient and Pearson’s correlation coefficient for the colocalization of the indicated marker with wild type or Q68L RAB10. Individual data points represent single cells imaged in 2–3 biological replicates; error bars indicate standard deviation. ∗*P* value < 0.01; ∗∗∗*P* value < 0.0007; ∗∗∗∗*P* value < 0.0001 (two-way ANOVA).

We next assessed the localization of RAB10 in HuH7 cells by immunofluorescence microscopy. Staining of *RAB10*-targeted cells confirmed the specificity of the RAB10 antibody (Fig. 3A). We observed colocalization of RAB10 with markers of the early endosome (EEA1), recycling endosome (RAB11), trans-Golgi network (TGN46), and endoplasmic reticulum (ER, PDI) (Fig. 3B), consistent with prior studies of RAB10 in other cell types (25, 31, 32). We quantified the relative distribution of RAB10 in these intracellular compartments and observed that RAB10 was sparsely distributed in EEA1-positive early endosomes and in the TGN46-positive trans-Golgi network. We also observed a large pool of RAB10 colocalized with RAB11-positive recycling endosomes and with the PDI-positive ER (Fig. 3C).

We next tested how the GTPase cycle of RAB10 affected its localization by comparison of wild-type, GTP-locked (Q68L), or GDP-locked (T23N) RAB10 expressed in a clonal cell line deleted for endogenous RAB10 (Fig. 3D). Consistent with previous reports (33, 34), we observed significantly decreased steady-state protein levels for the GDP-locked mutant by both immunoblotting (Fig. 3D) and immunofluorescence (Fig. 3E). In contrast, steady-state levels of the GTP-locked (Q68L) mutant were comparable to those of wild-type RAB10 (Fig 3D, E). In comparison to wild-type RAB10, GTP-locked RAB10 demonstrated increased colocalization with TGN46 and decreased colocalization with Rab11 (Fig. 3F, G). Together, these findings highlight the heterogeneous subcellular distribution of RAB10, its modulation by the GTPase cycle, and its potential to directly regulate the vesicular trafficking of LDLR and TFR.

### RAB10 depletion induces the redistribution of LDLR and TFR within subcellular compartments

**Fig. 4:**
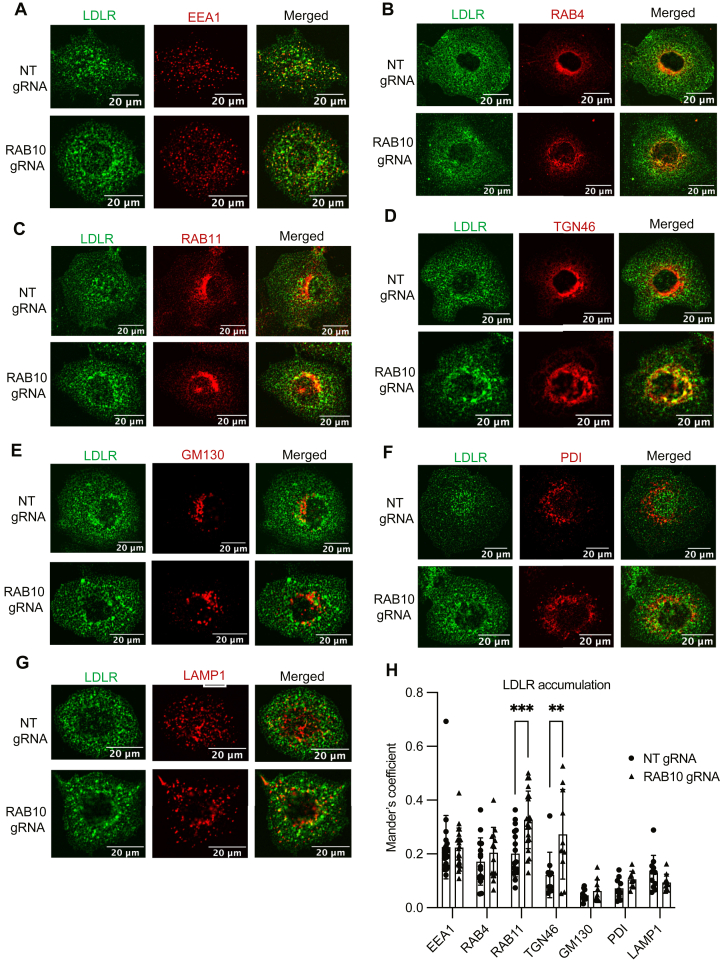
*RAB10* deletion alters LDLR intracellular distribution. A–G: HuH7 cells treated with either a *RAB10*-targeting gRNA or a nontargeting control (NT) were co-stained for LDLR and intracellular compartment markers EEA1 (A), RAB4 (B), RAB11 (C), TGN46 (D), GM130 (E), PDI (F), or LAMP1 (G). H: Mander’s overlap of LDLR with each indicated marker. Individual data points represent single cells imaged in 2–3 biological replicates; error bars indicate standard deviation. ∗∗ *P* value < 0.001; ∗∗∗*P* value < 0.0001 (two-way ANOVA). LDLR, LDL receptor; TFR, transferrin receptor.

By confocal imaging, we observed that a subset of RAB10 colocalized with both LDLR and TFR in intracellular punctae (supplemental Fig. S2A, B). We examined the impact of RAB10 depletion on the intracellular distribution of LDLR and TFR. Consistent with the cellular accumulation of LDLR and TFR in *RAB10*-targeted cells detected by immunoblotting and flow cytometry (Fig. 2A–C, F–H), immunofluorescence of *RAB10*-targeted cells revealed increased staining for both LDLR and TFR that remained distributed in punctae (Fig. 4). Colocalization analysis revealed the intracellular accumulation of LDLR to occur primarily in RAB11-positive recycling endosomes in RAB10-deleted cells (Fig. 4C, H), consistent with a role for RAB10 in receptor recycling, with no change in colocalization with the early endosomal marker EEA1(Fig. 4A, H), the early endosomal/rapid recycling marker RAB4 (Fig. 4B, H), the cis-Golgi marker GM130(Fig. 4E, H), the ER marker PDI(Fig. 4F, H), or the lysosomal marker Lamp1(Fig. 4G, H). Significant LDLR accumulation was also observed in the trans-Golgi network, as reflected by TGN46 colocalization (Fig. 4D, H).

**Fig. 5:**
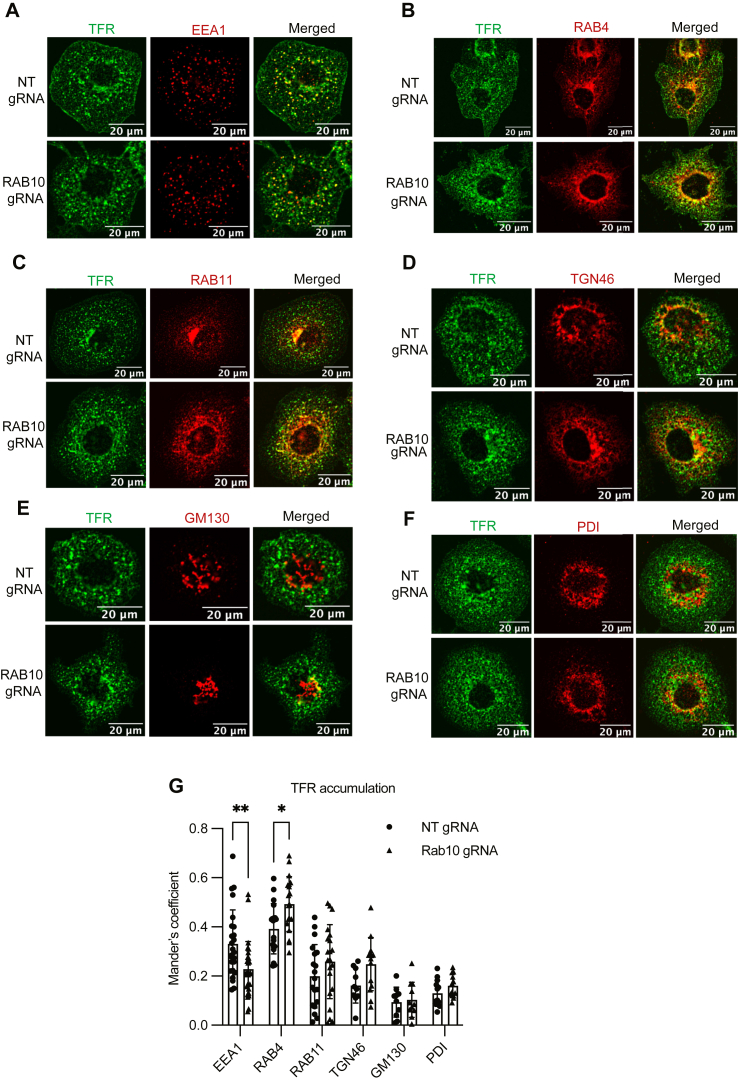
*RAB10* deletion alters intracellular distribution of TFR. A–F: HuH7 cells treated with either a *RAB10-*targeting gRNA or nontargeting control (NT) were co-stained for TFR and intracellular compartment markers EEA1 (A), RAB4 (B), RAB11 (C), TGN46 (D), GM130 (E), and PDI (F). G: Mander’s overlap of TFR with each indicated marker. Individual data points represent single cells imaged in 2–3 biological replicates; error bars indicate standard deviation. ∗*P* value < 0.01; ∗∗*P* value < 0.005 (two-way ANOVA). LDLR, LDL receptor; TFR, transferrin receptor.

A similar analysis of TFR redistribution in *RAB10*-targeted cells revealed a significant increase in colocalization with RAB4 (Fig. 5B, G) and a reduced colocalization with EEA1 (Fig. 5A, G). In contrast to LDLR, no significant increase in colocalization was observed for TFR with Rab11 and TGN46 (Fig. 5C, D, G). Similar to LDLR, no change in TFR colocalization was observed for markers of the cis-Golgi network (Fig. 5E, G) or the ER (Fig. 5F, G). These findings suggest that RAB10 depletion affects distribution of LDLR and TFR in distinct recycling compartments.

### RAB10 does not alter the kinetics of endocytosis for LDLR or TFR

**Fig. 6:**
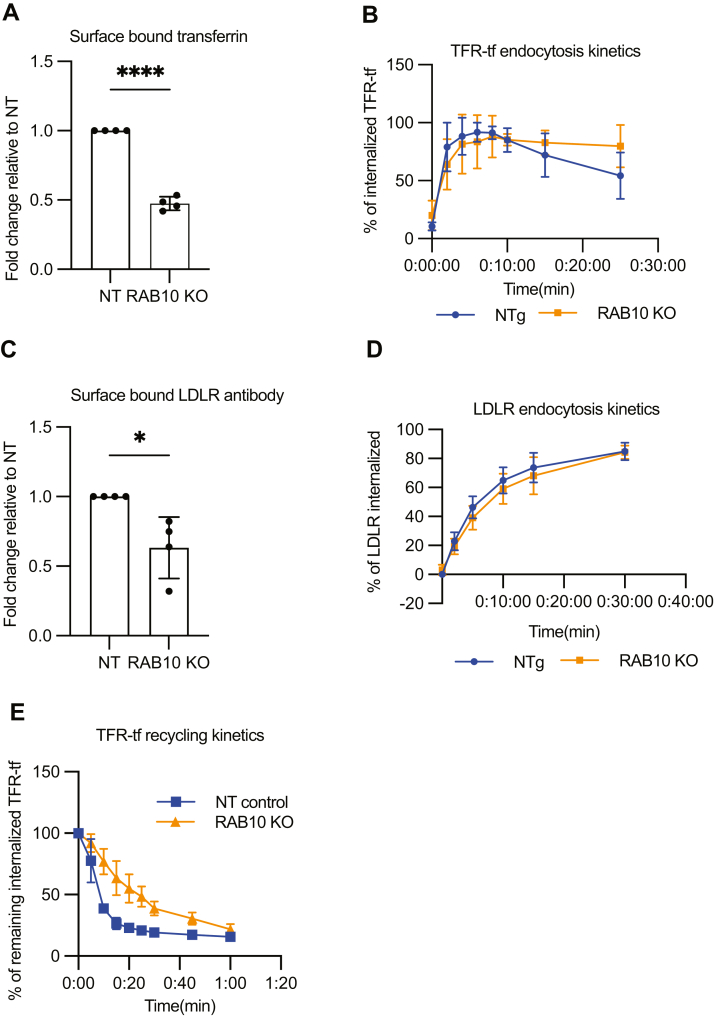
RAB10 regulates recycling but not endocytosis. A–B: HuH7 cells transduced with a *RAB10*-targeting gRNA or nontargeting gRNA control (NT) were assessed for surface binding of transferrin at time 0 (A) and at serial time points following endocytosis for internalization of surface-bound transferrin (B) by flow cytometry. C–D: Surface-exposed LDLR was labeled with fluorescent antibody and assayed for internalization after various time points by flow cytometry. E: TFR recycling was assayed by synchronizing cells with internalized transferrin and tracking the reduction in fluorescent signal at indicated time points by flow cytometry. Error bars represent standard deviation for four biologic replicates. ∗*P* value < 0.01; ∗∗∗∗*P* value < 0.0001 (Student’s *t-*test). LDLR, LDL receptor; TFR, transferrin receptor.

The redistribution of LDLR and TFR from the plasma membrane to endosomes in *RAB10*-targeted cells could be due to an influence on endocytosis or recycling. To distinguish between these possibilities, we first examined endocytosis of TFR in complex with fluorescently labeled transferrin in *RAB10*-targeted and control cells using a previously reported approach (21, 35). Briefly, cell-surface TFR was saturated with fluorescently conjugated transferrin, with samples cooled to 4°C to block endocytosis. Endocytosis was then induced by increasing the temperature to 37°C for various intervals, after which the temperature was again rapidly lowered to 4°C, and remaining surface-bound transferrin was removed by washing with acidic buffer. Internalized TFR–transferrin complex was then quantified by flow cytometry. At time zero, less fluorescent transferrin was bound to RAB10-depleted cells compared to control cells (Fig. 6A), consistent with the decreased surface TFR abundance in *RAB10*-targeted cells by flow cytometry (Fig. 2J). For the fluorescent transferrin that was bound to the cell surface, internalization was complete within 5–10 min, with no significant difference in the rate of endocytosis between RAB10-depleted and control cells (Fig. 6B).

In contrast to the TFR–transferrin complex, LDLR-LDL dissociates in the acidic environment of early endosomes with subsequent LDL degradation (22, 29), which limits the utility of fluorescent LDL to monitor endocytosis. We therefore used an antibody that recognizes an LDLR extracellular epitope to assay LDLR endocytosis kinetics (22, 23, 36). Cell-surface LDLR was first saturated with LDLR antibody at 4°C, with endocytosis then triggered by a temperature shift to 37°C. Samples taken at different time points were then quickly chilled to block further endocytosis, and the remaining surface LDLR–antibody complex was stained with fluorescently labeled secondary antibody, with the fraction of internalized LDLR antibody reflected by its protection from surface staining with secondary antibody. Consistent with decreased surface LDLR in RAB10-depleted cells, less fluorescent antibody was bound at time zero compared to control cells (Figs. 6C and 2E). Similar to TFR, no difference was observed in the rate of LDLR endocytosis between control and RAB10-depleted cells (Fig. 6D).

### RAB10 promotes the recycling of TFR

In our assay of TFR endocytosis (Fig. 6B), we noted a trend toward a decrease in cellular fluorescence after 10 min in control cells that was less pronounced in RAB10-depleted cells. This time frame is consistent with what would be expected for bulk recycling of endocytosed receptor from common endosomes (20, 21, 37, 38). We thus examined the recycling kinetics of TFR in response to RAB10 depletion. Cells were loaded with fluorescent transferrin at 37°C for 30 min, endocytosis was then blocked by cold treatment, and recycling was induced by shifting samples to 37°C for different chase periods. Samples of cells at specific time points were quickly chilled, and resurfaced TFR-transferrin was acid-washed. Remaining intracellular TFR-transferrin was then quantified by flow cytometry. *RAB10*-targeting resulted in a significant delay in TFR recycling, with 50% of the intracellular transferrin–TFR complexes recycled within 8 min for control cells compared to 15–20 min for RAB10-targeted cells (Fig. 6E).

## Discussion

Recycling of endocytosed membrane proteins to the cell surface plays an important role in maintaining the composition of the plasma membrane and the physiologic functions of the recycled proteins (39, 40, 41). Together with gene expression, protein secretion, and protein turnover, recycling regulates the steady-state level of a given receptor protein on the cell surface. The initial endocytosis of integral membrane proteins shares similar features, with receptors often releasing their ligands in the acidic lumen of early endosomes. After complex dissociation, receptors may then recycle back to the plasma membrane, either directly or via the endocytic recycling compartment and late recycling vesicles.

**Fig. 7:**
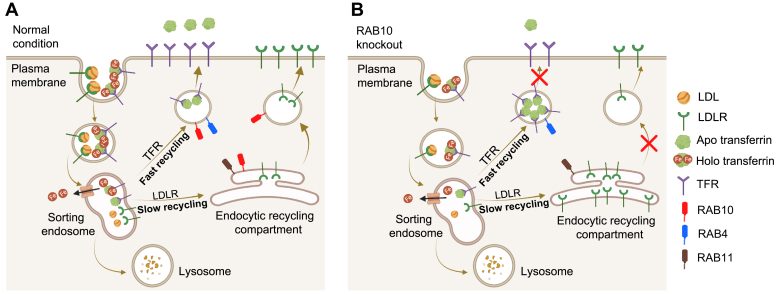
Differential effects of RAB10 deletion on LDLR and TFR recycling. A: LDL-bound LDLR and holo transferrin–bound TFR undergo clathrin-mediated endocytosis, upon which LDLR releases LDL and transferrin releases iron molecules within sorting endosome. LDLR is then transported via an endocytic recycling compartment (ERC) and recycled to the cell surface. The majority (80%–90%) of apo transferrin bound TFR is recycled through a fast-recycling route to the cell surface. B: Deletion of RAB10 results in a defect in trafficking of both fast and slow recycling vesicles, leading to accumulation of LDLR in RAB11-positive ERC and TFR in RAB4-positive fast recycling vesicles, resulting in reduced cell surface LDLR and TFR. Despite both LDLR and TFR accumulating within recycling compartments, only transferrin, which remains in complex with TFR, also accumulates in RAB10-deleted cells, as LDL dissociates from LDLR and undergoes degradation within lysosomes. LDLR, LDL receptor; TFR, transferrin receptor.

Rab GTPases have previously been reported to play broad roles in the regulation of vesicular trafficking. We recently identified the small GTPase RAB10 as a putative modifier of cellular LDL and transferrin uptake (13). In the current report, we confirmed the discordant effects of RAB10 on LDL and transferrin cellular accumulation, with the former decreased and the latter increased upon RAB10 depletion (Fig. 1B, C). Unexpectedly, in contrast to the opposing effects of RAB10 depletion on LDL and transferrin uptake, we observed similar effects on their corresponding receptors, LDLR and TFR. This discrepancy was likely due to the different fates of the two ligands following uptake, with LDL undergoing dissociation from LDLR while transferrin remains in complex with TFR during recycling until its release extracellularly. This process is summarized schematically in Fig. 7.

Several lines of evidence support a model in which RAB10 promotes the recycling of both LDLR and TFR. First, RAB10 depletion caused a decrease in the amount of both receptors on the cell surface without a corresponding decrease in gene expression (Fig. 2). Second, RAB10 depletion also caused an intracellular accumulation of both receptors in recycling organelles consistent with a delay in their plasma membrane recycling (Figs. 4H–I and 5G, H). Third, a subpopulation of RAB10 was found to colocalize with both receptors and with recycling endosomes. Fourth, the association of GTP-locked RAB10 mutant with recycling endosomes was decreased, consistent with this active form accelerating the anterograde transport of cargo vesicles out of this compartment. Finally, kinetic experiments confirmed a delay in TFR recycling to the plasma membrane (Fig. 6D).

Previous studies have revealed heterogeneity in the recycling of different receptors, with some, including LDLR, transported along a RAB11-mediated slow recycling pathway involving the endocytic recycling compartment, while others, including TFR, utilize both RAB11- and RAB4-mediated rapid recycling pathways (20, 42, 43). Our results implicate RAB10 in both pathways, as we observed LDLR accumulation in RAB11-positive punctae and TFR accumulation in RAB4-positive punctae upon RAB10 depletion.

Small GTPases function as molecular switches, cycling between a GDP-bound inactive state and GTP-bound active state that mediates recruitment of effector proteins to membranes. Intriguingly, GTP-bound RAB10 has previously been demonstrated to mediate the insulin-stimulated transport of GLUT4-containing vesicles to the plasma membrane via its recruitment of the exocyst membrane tethering complex (44). Our prior screen of LDL uptake modifiers likewise identified several exocyst components including *EXOC1, EXOC2, EXOC3, EXOC4, EXOC7,* and *EXOC8* that phenocopied *RAB10*, with depletion of either protein resulting in decreased LDL uptake and increased transferrin accumulation. Association of RAB10 with the exocyst complex has also been reported in renal epithelial cells (45). Taken together, these findings suggest that RAB10 may promote the recycling of LDLR and TFR through the recruitment of the exocyst to recycling vesicles. The previously reported CRISPR screen for modifiers of LDL uptake (13) also identified RABIF (Rab interacting factor), a guanine nucleotide exchange factor that stimulates GDP release from various Rab GTPases including RAB10, and which has also been shown to stabilize RAB10 (46). This screen also identified STX4, a SNARE protein that facilitates docking and fusion of transport vesicle with the cell membrane and has been similarly implicated in the fusion of GLUT4 vesicles with the plasma membrane (47). A recent study based on published proteomic data and CRISPR/Cas9 screens also identified a correlation between RAB10 and STX4 (48). Taken together, these findings suggest that RAB10, the exocyst, and STX4 may work together to coordinate the trafficking, tethering, and fusion of LDLR and TFR-containing recycling vesicles, similar to their role in GLUT4 vesicular trafficking.

RAB10 has also been implicated in diverse areas of membrane trafficking in different cell types, including formation of noncanonical macropinosome tubules in macrophages (49), vesicle transportation from early endosome to recycling endosome in *C. elegans* (31), and Golgi to plasma membrane transport in macrophages (32). Consistent with this wide range of functions, RAB10 has been localized to multiple subcellular compartments in different cell types including the endoplasmic reticulum, trans Golgi network, early endosomes, recycling endosomes, phagosomes, and primary cilia (25, 31, 32, 50, 51, 52). We also observed significant subpopulations of RAB10 in several subcellular compartments. In further support of the breadth of cellular functions for RAB10, germline deletion of RAB10 in mice results in embryonic lethality (53). This latter observation limits the direct confirmation of our current findings in an in vivo mouse model and, together with our demonstration that multiple receptors depend on RAB10 for recycling, suggests that the potential for RAB10-mediated LDLR recycling as a therapeutic target is likely to be limited by substantial off-target effects.

## Data availability

All data are contained within the manuscript.

## Supplemental data

This article contains supplemental data.

## Conflicts of interest

The authors declare that they have no conflicts of interest with the contents of this article.

## Supplemental data

Figure S1 RAB10 regulates the ratio of surface to total LDLR and TFR. A: Ratio of surface to total LDLR abundance for HuH7 cells treated with a gRNA targeting *RAB10* or a nontargeting (NT) control. ∗∗∗ *P* value <0.0002 (Student’s *t*-test). B: Ratio of surface to total LDLR abundance for wild-type HuH7 cells and HuH7 cells overexpressing wild-type or the Q68L mutant of RAB10. ∗∗*P* value = 0.0017 (one-way ANOVA). C: Ratio of surface to total TFR abundance for HuH7 cells treated with a *RAB10*-targeting gRNA or NT control. ∗∗∗∗ *P* value <0.0001 (Student’s *t*-test). Individual data points represent biologic replicates, and error bars indicate standard deviation. LDLR, LDL receptor; TFR, receptor for transferrin.

Figure S2 RAB10 colocalizes with LDLR and TFR. Immunofluorescence of HuH7 cells (A, C) with Pearson’s correlation coefficient and Mander’s overlap coefficient (B, D) for colocalization between RAB10 and LDLR (A, B) or TFR (C, D). Individual data points represent individual cells imaged in three biologic replicates; error bars indicate standard deviation. LDLR, LDL receptor; TFR, receptor for transferrin.
