# Supplementary figures and images for: The small GTPase RAB10 regulates endosomal recycling of the LDL receptor and transferrin receptor in hepatocytes
Source: J Lipid Res. 2022 Jun 24;63(8):100248. doi: 10.1016/j.jlr.2022.100248 (PMC9305350; doi:10.1016/j.jlr.2022.100248)

**A**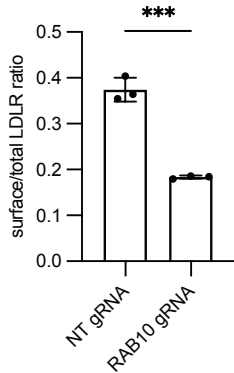**B**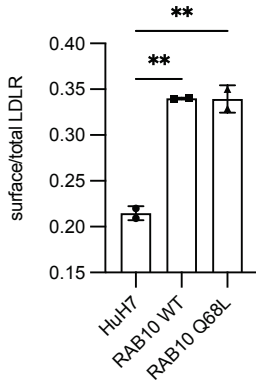**C**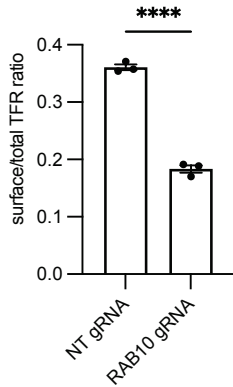

Supplement: Figure S1 — RAB10 regulates the ratio of surface to total LDLR and TFR. A: Ratio of surface to total LDLR abundance for HuH7 cells treated with a gRNA targeting RAB10 or a nontargeting (NT) control. ∗∗∗ P value <0.0002 (Student’s t-test). B: Ratio of surface to total LDLR abundance for wild-type HuH7 cells and HuH7 cells overexpressing wild-type or the Q68L mutant of RAB10. ∗∗P value = 0.0017 (one-way ANOVA). C: Ratio of surface to total TFR abundance for HuH7 cells treated with a RAB10-targeting gRNA or NT control. ∗∗∗∗ P value <0.0001 (Student’s t-test). Individual data points represent biologic replicates, and error bars indicate standard deviation. LDLR, LDL receptor; TFR, receptor for transferrin. [file mmc1.pdf]

**A**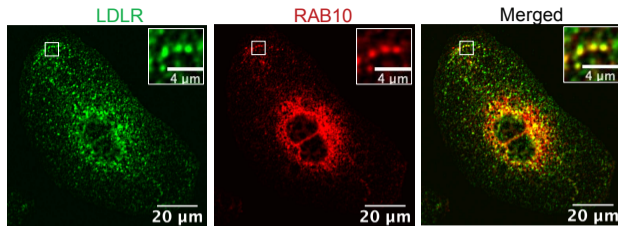**B**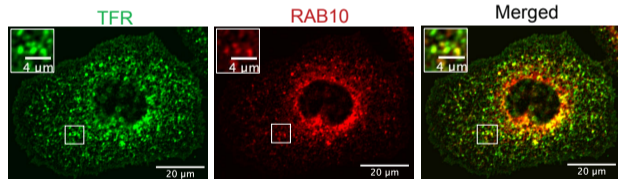**C**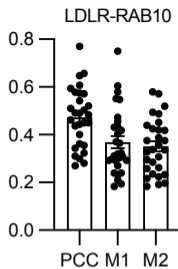**D**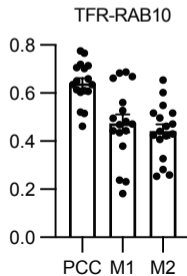

Supplement: Figure S2 — RAB10 colocalizes with LDLR and TFR. Immunofluorescence of HuH7 cells (A, C) with Pearson’s correlation coefficient and Mander’s overlap coefficient (B, D) for colocalization between RAB10 and LDLR (A, B) or TFR (C, D). Individual data points represent individual cells imaged in three biologic replicates; error bars indicate standard deviation. LDLR, LDL receptor; TFR, receptor for transferrin. [file mmc2.pdf]
